# Validation of the prognostic value of NF-κB p65 in prostate cancer: A retrospective study using a large multi-institutional cohort of the Canadian Prostate Cancer Biomarker Network

**DOI:** 10.1371/journal.pmed.1002847

**Published:** 2019-07-02

**Authors:** Andrée-Anne Grosset, Véronique Ouellet, Christine Caron, Gabriela Fragoso, Véronique Barrès, Nathalie Delvoye, Mathieu Latour, Armen Aprikian, Alain Bergeron, Simone Chevalier, Ladan Fazli, Neil Fleshner, Martin Gleave, Pierre Karakiewicz, Louis Lacombe, Jean-Baptiste Lattouf, Theodorus van der Kwast, Dominique Trudel, Anne-Marie Mes-Masson, Fred Saad, for the Canadian Prostate Cancer Biomarker Network

**Affiliations:** 1 Centre de recherche du Centre hospitalier de l’Université de Montréal, Centre hospitalier de l’Université de Montréal, Montreal, Quebec, Canada; 2 Institut du cancer de Montréal, Montreal, Quebec, Canada; 3 Research Institute of the McGill University Health Centre, McGill University Health Centre, Montreal, Quebec, Canada; 4 Axe Oncologie, Centre de recherche du Centre hospitalier universitaire de Québec–Université Laval, Centre hospitalier universitaire de Québec–Université Laval, Quebec, Quebec, Canada; 5 Centre de recherche sur le cancer, Université Laval, Quebec, Quebec, Canada; 6 Département de chirurgie, Université Laval, Quebec, Quebec, Canada; 7 Vancouver Prostate Centre, Vancouver, British Columbia, Canada; 8 University Health Network, Toronto, Ontario, Canada; Case Western Reserve Unversity, UNITED STATES

## Abstract

**Background:**

The identification of patients with high-risk prostate cancer (PC) is a major challenge for clinicians, and the improvement of current prognostic parameters is an unmet clinical need. We and others have identified an association between the nuclear localization of NF-κB p65 and biochemical recurrence (BCR) in PC in small and/or single-centre cohorts of patients.

**Methods and findings:**

In this study, we accessed 2 different multi-centre tissue microarrays (TMAs) representing cohorts of patients (Test-TMA and Validation-TMA series) of the Canadian Prostate Cancer Biomarker Network (CPCBN) to validate the association between p65 nuclear frequency and PC outcomes. Immunohistochemical staining of p65 was performed on the Test-TMA and Validation-TMA series, which include PC tissues from patients treated by first-line radical prostatectomy (*n* = 250 and *n* = 1,262, respectively). Two independent observers evaluated the p65 nuclear frequency in digital images of cancer tissue and benign adjacent gland tissue. Kaplan–Meier curves coupled with a log-rank test and univariate and multivariate Cox regression models were used for statistical analyses of continuous values and dichotomized data (cutoff of 3%). Multivariate analysis of the Validation-TMA cohort showed that p65 nuclear frequency in cancer cells was an independent predictor of BCR using continuous (hazard ratio [HR] 1.02 [95% CI 1.00–1.03], *p* = 0.004) and dichotomized data (HR 1.33 [95% CI 1.09–1.62], *p* = 0.005). Using a cutoff of 3%, we found that this biomarker was also associated with the development of bone metastases (HR 1.82 [95% CI 1.05–3.16], *p* = 0.033) and PC-specific mortality (HR 2.63 [95% CI 1.30–5.31], *p* = 0.004), independent of clinical parameters. BCR-free survival, bone-metastasis-free survival, and PC-specific survival were shorter for patients with higher p65 nuclear frequency (*p* < 0.005). As the small cores on TMAs are a limitation of the study, a backward validation of whole PC tissue section will be necessary for the implementation of p65 nuclear frequency as a PC biomarker in the clinical workflow.

**Conclusions:**

We report the first study using the pan-Canadian multi-centre cohorts of CPCBN and validate the association between increased frequency of nuclear p65 frequency and a risk of disease progression.

## Abstract 2

Fred Saad and colleagues reveal the prognostic association between nuclear NF-κB p65 expression and prostate cancer outcomes.

## Author summary

**Why was this study done?:**

- In Canada and the US, prostate cancer is the most commonly diagnosed cancer in men.
- Identifying patients with aggressive prostate cancer is crucial for the choice of treatment to increase survival.
- Previously, we and others showed that the localization of a biomarker, nuclear factor kappa B (NF-κB) p65, in the nucleus of prostate cancer cells allows the identification of patients with aggressive prostate cancer. More specifically, the nuclear expression of NF-κB p65 is associated with cancer recurrence.

**What did the researchers do and find?:**

- To further validate our findings in a larger, multi-centre cohort of patients, we took advantage of the Canadian Prostate Cancer Biomarker Network (CPCBN) tissue microarrays that include a test (*n* = 250) and a validation (*n* = 1,262) series built with radical prostatectomy specimens from prostate cancer patients.
- We showed that NF-κB p65 in the nucleus of prostate cancer cells was associated with all evaluated prostate cancer endpoints (biochemical recurrence, development of bone metastases, and prostate-cancer-specific death).

**What do these findings mean?:**

- We validated the association between NF-κB p65 nuclear frequency and more aggressive prostate cancer.
- Nuclear frequency of NF-κB p65 should help to better identify patients with a higher risk of disease progression, and this could impact patient management.
- For the implementation of this biomarker in the clinical workflow, the investigation of nuclear frequency of NF-κB p65 in whole diagnostic samples will be important.

## Introduction

Prostate cancer (PC) is the most commonly diagnosed cancer in Canadian men [1]. In men with high-risk PC, progression of the disease will lead to biochemical recurrence (BCR), distant metastases, and disease-specific mortality. Up to now, patient prognosis has been based on 3 parameters: preoperative prostate-specific antigen (PSA) level, stage, and Gleason score [2]. However, these are not always sufficient for accurate stratification of patients. The identification of high-risk PC patients is a major challenge for clinicians, and failure to correctly identify these cases leads to disease progression that does not receive the most appropriate management. To more accurately predict PC prognosis, rigorous validation and clinical implementation of new prognostic markers are required [3].

The extensively studied nuclear factor kappa B (NF-κB) pathway is involved in the regulation of inflammation and the immune response [4], and more recently demonstrated its importance in cancer development [5]. Homo- or heterodimers of 5 subunits (p65, c-rel, RelB, p50, and p52) are implicated in the NF-κB signalling pathway to induce the expression of target genes. In the canonical pathway, the inactive form of the p65/p50 dimer is associated with the inhibitor IκB and is retained in the cytoplasm. Once phosphorylated, IκB releases the p65/p50 dimer and is degraded by the proteasome while the dimer translocates to the nucleus, allowing the transcription of target genes. The activation of the NF-κB signalling pathways can result in the progression of several types of cancer, including PC [6].

Previously, we identified the nuclear localization of p65 as a prognostic biomarker in PC patients [7]. We first showed with immunohistochemical staining that nuclear expression of p65 in positive surgical margins of tissue was associated with BCR in a small cohort of 42 patients [8]. This association was also observed in PC tissues using tissue microarrays (TMAs) comprised of specimens from 63 patients [9]. Subsequently, we used a large independent cohort of 1,826 PC patients to validate the relation between nuclear translocation of p65 and increased risk for BCR [10]. More recently, a study in a cohort of 200 PC patients confirmed the frequency of nuclear p65 expression as a prognostic indicator of BCR risk with an immunofluorescence-based approach [11]. These observations were also reported independently by other groups [12–14].

Despite their identification of p65 nuclear expression as a strong predictor of BCR, these studies evaluated p65 in small or single-centre cohorts. To represent the entire population and avoid site-specific biases, multi-institutional cohorts are needed. Overall, several studies have been conducted on promising prognostic biomarkers, but none have been added to current clinical parameters for PC [15]. One of the mandates of the Canadian Prostate Cancer Biomarker Network (CPCBN) is to validate such prognostic biomarkers and accelerate their integration into the clinical workflow to improve PC patient management. For this purpose, the TMA platform of CPCBN includes 2 independent multi-institutional cohorts of PC patients treated by first-line radical prostatectomy (RP) with complete clinical data [16]. The Test-TMA cohort, comprising 250 PC patients, evaluates the prognostic ability of a biomarker. Following conclusive results, the biomarker is next analyzed on specimens from the 1,262 PC patients of the Validation-TMA to identify high-risk PC patients.

In the present study, we validated the nuclear expression of p65 as an independent predictor of BCR in these 2 independent multi-institutional cohorts of PC patients. This is to our knowledge the first study to show the prognostic value of p65 nuclear frequency for the development of bone metastases and PC-specific death.

## Methods

### Patients and CPCBN TMAs

The TMAs of the CPCBN are composed of RP specimens from 2 independent cohorts of 250 and 1,262 PC patients who agreed to participate in the biobank of 1 of 5 Canadian institutions (Centre de recherche du Centre hospitalier de l’Université de Montréal, Research Institute of the McGill University Health Centre, Centre de recherche du Centre hospitalier universitaire de Québec–Université Laval, University Health Network, and Vancouver Prostate Centre). All patients signed an informed consent for the use of their prostate tissue samples in research. Each biobank received the approval from their local ethics review board for inclusion of prostate tissue samples (*n* = 300 per site) in the CPCBN resource. Treatment-naïve RP specimens were collected from 1990 to 2011. The first cohort of 250 specimens (*n* = 50 per site) was named the ‘Test-TMA series’, and the second cohort of 1,262 samples (*n* ≈ 250 per site) was labelled as the ‘Validation-TMA series’ [16]. For each patient, 0.6-mm cores (3–4 of tumor tissue and 1–2 of benign adjacent tissue) were punctured from formalin-fixed paraffin-embedded blocks and transferred to receiver blocks.

### Immunohistochemistry

Four-micrometre-thick sections of each TMA block were subjected to antigen retrieval in Cell Conditioning 1 (#950–124, Ventana Medical Systems, Tucson, AZ, US) for 90 minutes and then stained using the BenchMark XT autostainer (Ventana Medical Systems). Pre-diluted anti-NF-kB p65 mouse monoclonal antibody (sc-8008 [F-6], Santa Cruz Biotechnology, Santa Cruz, CA, US) at 1:100 was manually added to the slides and incubated at 37°C for 60 minutes. Antibody binding was revealed using the ultraView Universal DAB Detection Kit (#760–500, Ventana Medical Systems). Counterstaining was achieved using hematoxylin and bluing reagents (#760–2021 and #760–2037, Ventana Medical Systems). Tissues were dehydrated and mounted using Sub-X Mounting Medium (Leica Microsystems, Concord, ON, Canada). All sections were scanned using a VS-110 microscope with a 20× 0.75 NA objective and a resolution of 0.3225 μm (Olympus Canada, Richmond Hill, ON, Canada).

### Quantification of nuclear p65 expression

The frequency (0%–100%) of epithelial cells with nuclear p65 expression in benign and tumor cores was assessed by 2 observers using digitalized images. Interclass correlation of the scoring for each core between the 2 observers was 0.88. When more than 1 core per patient was evaluated, the average p65 nuclear frequency for each patient (benign adjacent tissue and tumor) was used for subsequent analyses.

### Statistics

Statistical analyses were performed with SPSS version 23.0 (SPSS, Chicago, IL, US). Interclass comparisons were performed to evaluate the agreement between the 2 observers. The correlation with clinicopathological parameters was estimated with a non-parametric Spearman correlation test. The analysis plan was to evaluate the association of p65 nuclear frequency with PC patient clinical endpoints, which included the BCR, the development of bone metastases, and PC-specific mortality. The cutoff applied for the dichotomization of the data was defined by the median frequency of nuclear p65 expression (3%) in the Test-TMA series. This threshold was then applied to the Validation-TMA series. BCR-free, bone-metastasis-free, and PC-specific survival curves were plotted using the Kaplan–Meier estimator, and the log-rank test was used to evaluate significant differences. The univariate and multivariate proportional hazard models (Cox regression) were used to estimate the hazard ratios (HRs) for nuclear p65 frequency. For multivariate analyses, the serum PSA level prior to RP, the pathological staging of the primary tumor (pT2, pT3, pT4), the Gleason score category (6, 7 [3+4], 7 [4+3], 8+), and the margin status (negative/positive) were included in the model. In the rare occasion where clinical data were missing, the case was withdrawn from the analyses. Results were considered statistically significant at *p*-values below 0.05.

The STROBE checklist is provided in S1 Table.

## Results

### The nuclear localization of p65 in PC and increased risk of BCR

The multi-institutional CPCBN cohorts and TMAs have been previously characterized [16]. Pathology review and grading of each core had been performed and showed that Gleason grading of TMA cores agreed with RP specimen grades [17]. Nuclear p65 expression in the CPCBN TMAs was assessed in 2 distinct steps. First, we attained access to the Test-TMA series, a cohort of 250 patients. Next, our results were reviewed by the CPCBN committee for approval of access to the second cohort of 1,262 patients, the TMA-Validation series. Clinicopathological characteristics of PC patients in each cohort are presented in Table 1.

**Table 1:** Clinicopathological characteristics of prostate cancer patients from the 2 Canadian Prostate Cancer Biomarker Network cohorts.

Parameter	Test-TMA	Validation-TMA
Number of patients	250	1,262
Age in years at diagnosis	62 (9)	62 (9)
Follow-up in months	113 (62)	120 (71)
Biochemical recurrence	77 (31)	434 (34)
Biochemical recurrence type		
PSA > 0.2 ng/ml and rising	54 (22)	264 (21)
Failed RP	16 (6)	85 (7)
PSA followed by a decision of treatment	7 (3)	85 (7)
Bone metastases	11 (4)	54 (4)
Castrate resistant	13 (5)	61 (5)
Death		
Prostate-cancer-specific	4 (2)	36 (3)
Other cause	17 (7)	119 (9)
Overall	21 (8)	155 (12)
RP Gleason score		
≤3+3	64 (26)	392 (31)
3+4	104 (42)	499 (39)
4+3	42 (17)	188 (15)
≥4+4	36 (14)	175 (14)
Undetermined	4 (2)	8 (1)
Pathological staging of the primary tumor		
pT2	171 (68)	788 (62)
pT3	77 (31)	453 (36)
pT4	2 (1)	21 (2)
Margin positive (invasive carcinoma involvement)	91 (36)	418 (33)

Data given as number (percent) or median (standard error).

PSA, prostate-specific antigen; RP, radical prostatectomy.

**Fig 1:**
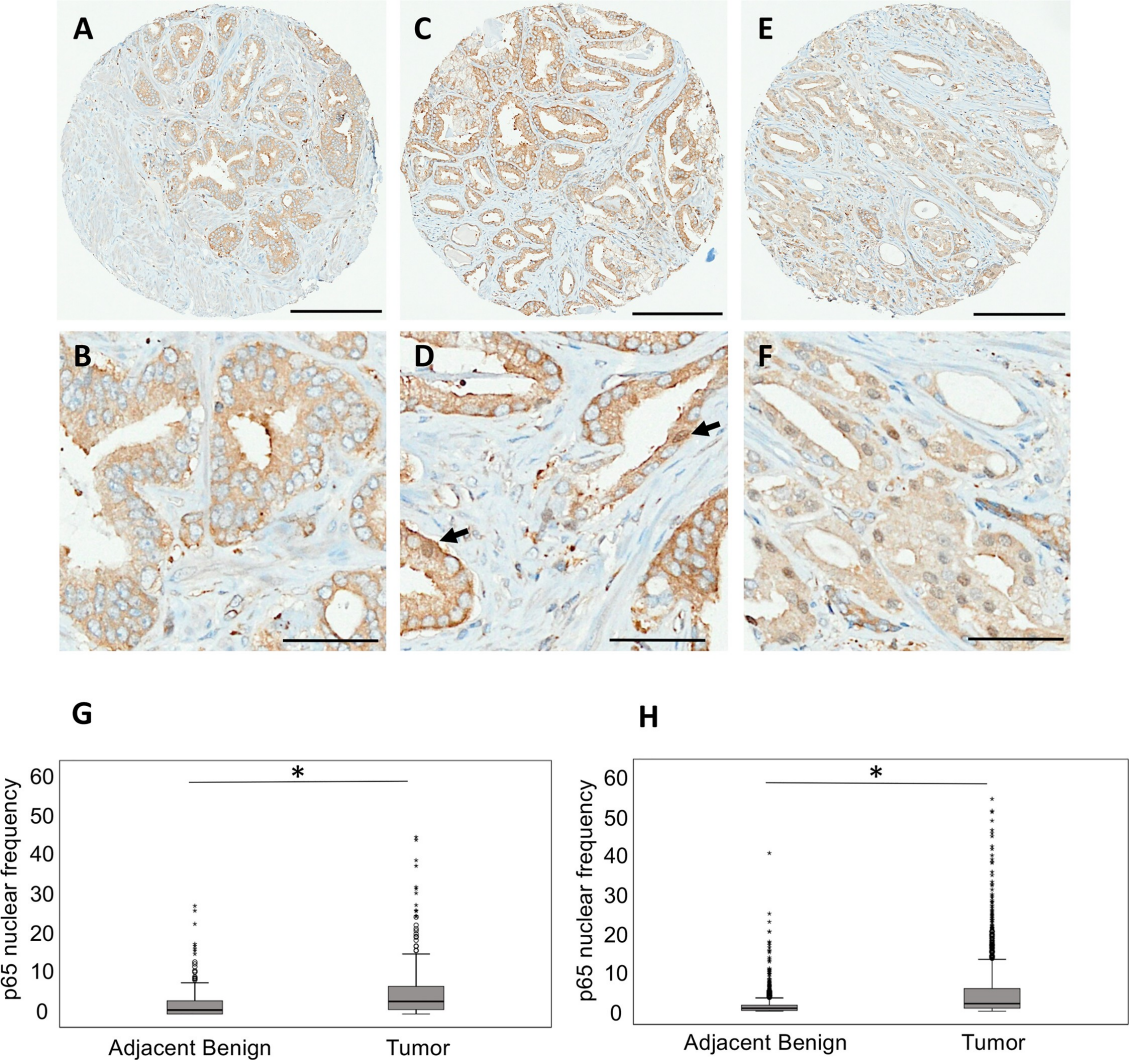
Nuclear localization of p65 in prostate cancer tissues. Representative images of p65 immunostaining on TMA cores of prostate cancer. (A, B) Negative nuclear staining (negative), (C, D) ≤3% positive nuclear staining (low expression), and (E, F) >3% positive nuclear staining (high expression). Arrows in (D) indicate rare positive nuclei. Scale bars, 200 μm (A, C, E) and 50 μm (B, D, F). Box plot representation of the nuclear frequency of p65 in benign adjacent and tumor cores of the Test-TMA (G) and Validation-TMA (H). *p < 0.001 (Mann–Whitney U test).

Immunohistochemical staining of both TMA series showed variable levels of nuclear p65 expression in PC specimens, ranging from 0% to 100% of epithelial cells with positive staining. Median frequency of nuclear p65 expression in tumor cores was 3%. Representative images of negative (0%), low (>0% and ≤3%), and high (>3%) expression are presented in Fig 1. We observed a statistically significantly higher level of p65 nuclear frequency in tumor cores compared to benign adjacent cores in both the Test-TMA and the Validation-TMA (Fig 1G and 1H, respectively).

**Fig 2:**
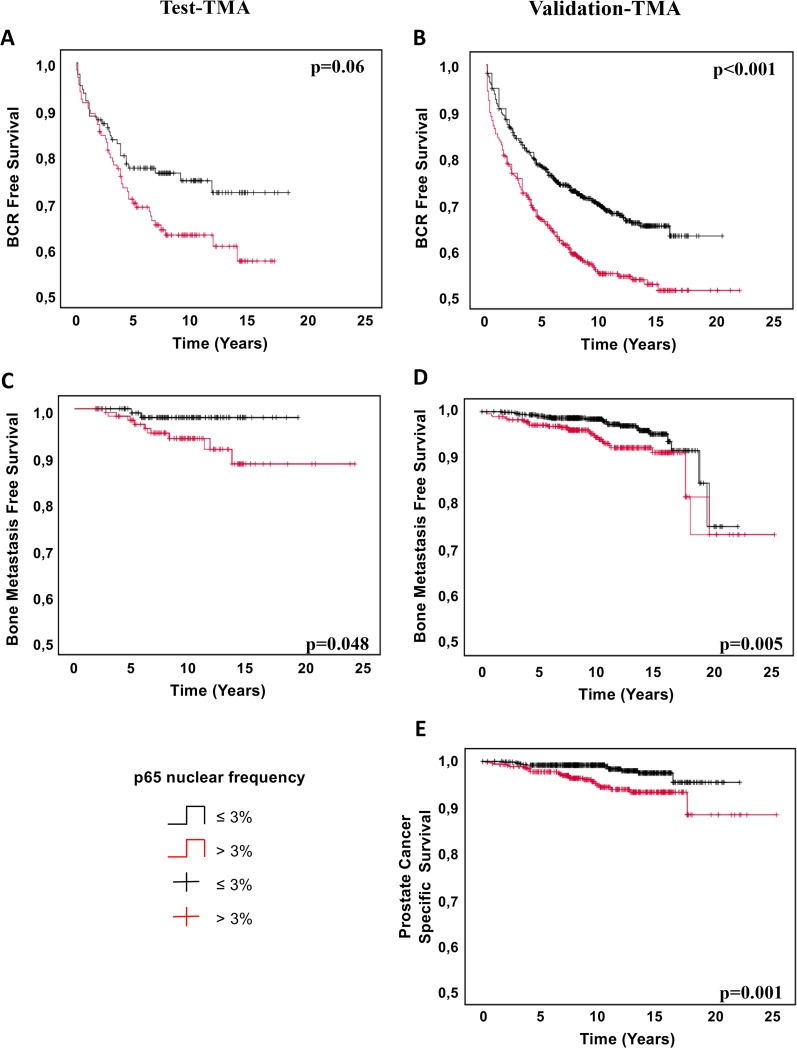
Association of p65 nuclear frequency with prostate cancer patient outcome. Kaplan–Meier curves for (A, B) BCR-free survival, (C, D) bone-metastasis-free survival, and (E) prostate-cancer-specific survival, stratified by negative/low (0%–3%) and high (>3%) p65 nuclear expression in prostate cancer cells. *p*-Values are calculated using the log-rank test. *p* < 0.05 is considered significant. BCR, biochemical recurrence.

Correlation of p65 nuclear frequency with clinical characteristics was evaluated as continuous or dichotomized data. Threshold was based on the median nuclear frequency of 3% in tumor cores and 1% in benign adjacent cores in the Test-TMA series. The same threshold was applied to the Validation-TMA series. The Test-TMA cohort contained 128 PC patients with >3% positive nuclear expression of p65 in tumors compared to 456 PC patients of the Validation-TMA cohort. Kaplan–Meier survival curves show that nuclear p65 expression tended to be associated with an increased risk of BCR in the Test-TMA cohort (*p* = 0.06) (Fig 2A), and this association was highly significant in the Validation-TMA cohort (*p* < 0.001) (Fig 2B). Univariate Cox regression analysis demonstrated that all known predictors of BCR (preoperative PSA level, pathological staging, RP Gleason score, and margin status) were statistically significant in both cohorts. Although univariate regression analyses of both continuous (HR 1.02 [95% CI 1.00–1.05], *p* = 0.099) and dichotomized (HR 1.54 [95% CI 0.98–2.44], *p* = 0.063) data of p65 nuclear frequency in tumors did not reach significance in the Test-TMA series (Table 2), a strong association was observed for continuous (HR 1.03 [95% CI 1.02–1.04], *p* < 0.001) and dichotomized (HR 1.60 [95% CI 1.32–1.94], *p* < 0.001) data in the Validation-TMA series (Table 3). In a multivariate analysis, nuclear p65 was an independent prognostic parameter for BCR in both continuous (HR 1.02 [95% CI 1.00–1.03], *p* = 0.004) and dichotomized (HR 1.33 [95% CI 1.09–1.62], *p* = 0.005) data in the Validation-TMA series (Table 3). In contrast, nuclear p65 in benign adjacent tissues was not associated with BCR (Tables 2 and 3).

**Table 2:** Univariate and multivariate Cox regression analyses predicting biochemical recurrence in the Test-TMA series.

Parameter	Univariate analysis		Multivariate analysis	
	HR (95% CI)	*p-*Value	HR (95% CI)	*p-*Value
Preoperative PSA	1.06 (1.04–1.09)	**<0.001**	1.03 (1.01–1.05)	**0.007**
pT	4.67 (3.13–6.97)	**<0.001**	2.89 (1.79–4.69)	**<0.001**
RP Gleason score	2.03 (1.63–2.54)	**<0.001**	1.65 (1.29–2.09)	**<0.001**
Margin status	2.39 (1.52–3.77)	**<0.001**	1.41 (0.87–2.28)	0.163
*Benign adjacent tissue*				
p65 continuous	1.02 (0.97–1.07)	0.356	1.02 (0.98–1.07)	0.300
p65 >3% versus ≤3%	1.14 (0.73–1.78)	0.567	1.35 (0.85–2.15)	0.205
*Tumor tissue*				
p65 continuous	1.02 (1.00–1.05)	0.099	1.02 (0.99–1.05)	0.216
p65 >3% versus ≤3%	1.54 (0.98–2.44)	0.063	1.29 (0.80–2.07)	0.296

Significant *p*-values given in bold.

95% CI, 95% confidence interval; HR, hazard ratio; PSA, prostate-specific antigen; pT, pathological staging of the primary tumor; RP, radical prostatectomy.

**Table 3:** Univariate and multivariate Cox regression analyses predicting biochemical recurrence in the Validation-TMA series.

Parameter	Univariate analysis		Multivariate analysis	
	HR (95% CI)	*p-*Value	HR (95% CI)	*p-*Value
Preoperative PSA	1.03 (1.03–1.04)	**<0.001**	1.02 (1.01–1.03)	**<0.001**
pT	2.60 (2.20–3.07)	**<0.001**	1.44 (1.18–1.74)	**<0.001**
RP Gleason score	1.95 (1.78–2.13)	**<0.001**	1.67 (1.51–1.85)	**<0.001**
Margin status	2.36 (1.96–2.85)	**<0.001**	1.71 (1.40–2.10)	**<0.001**
*Benign adjacent tissue*				
p65 continuous	0.99 (0.95–1.03)	0.645	1.00 (0.97–1.04)	0.779
p65 >3% versus ≤3%	1.06 (0.86–1.30)	0.575	1.13 (0.92–1.40)	0.249
*Tumor tissue*				
p65 continuous	1.03 (1.02–1.04)	**<0.001**	1.02 (1.00–1.03)	**0.004**
p65 >3% versus ≤3%	1.60 (1.32–1.94)	**<0.001**	1.33 (1.09–1.62)	**0.005**

Significant *p*-values given in bold.

95% CI, 95% confidence interval; HR, hazard ratio; PSA, prostate-specific antigen; pT, pathological staging of the primary tumor; RP, radical prostatectomy.

### Development of bone metastases predicted by nuclear p65 expression

The progression of PC leads to the spread of the disease, particularly to bones [18]. To evaluate the prognostic significance of the nuclear localization of p65, we assessed its association with the development of bone metastases. In univariate and multivariate Cox regression analyses, pathological staging and RP Gleason score showed an association with bone metastases in both TMA series (Tables 4 and 5). Although significant in univariate analysis, preoperative PSA level did not remain significant in the multivariate analysis. Univariate analysis on the Test-TMA cohort showed that the continuous value of p65 nuclear frequency was significantly associated with bone metastases (HR 1.06 [95% CI 1.01–1.11], *p* = 0.023); dichotomized data of nuclear p65 almost reached a significant association (HR 4.14 [95% CI 0.89–19.19], *p* = 0.069), with an HR of 4.14. The Validation-TMA series confirmed that p65 nuclear expression was a strong predictor of bone metastasis development using both continuous (HR 1.04 [95% CI 1.01–1.06], *p* = 0.003) and dichotomized (HR 2.13 [95% CI 1.23–3.66], *p* = 0.007) data. This association remained significant when clinical parameters (Gleason score and pathological staging) were included in the model using the dichotomized data (HR 2.63 [95% CI 1.30–5.31], *p* = 0.033). Kaplan–Meier estimates also demonstrated that bone-metastasis-free survival was shorter for PC patients with a high p65 nuclear localization in both TMA series (log-rank *p* = 0.048 for Test-TMA and *p* = 0.005 for Validation-TMA) (Fig 2C and 2D).

**Table 4:** Univariate Cox regression analysis predicting bone metastasis development in the Test-TMA series.

Parameter	Univariate analysis	
	HR (95% CI)	*p-*Value
Preoperative PSA	1.05 (1.00–1.10)	0.051
pT	8.40 (3.04–23.16)	**<0.001**
RP Gleason score	3.16 (1.60–6.24)	**0.001**
Margin status	2.62 (0.76–9.01)	0.125
*Benign adjacent tissue*		
p65 continuous	0.98 (0.83–1.15)	0.803
p65 >3% versus ≤3%	1.30 (0.40–4.29)	0.663
*Tumor tissue*		
p65 continuous	1.06 (1.01–1.11)	**0.023**
p65 >3% versus ≤3%	4.14 (0.89–19.19)	0.069

Significant *p*-values given in bold.

95% CI, 95% confidence interval; HR, hazard ratio; PSA, prostate-specific antigen; pT, pathological staging of the primary tumor; RP, radical prostatectomy.

**Table 5:** Univariate and multivariate Cox regression analysis predicting bone metastasis development in the Validation-TMA series.

Parameter	Univariate analysis		Multivariate analysis	
	HR (95% CI)	*p-*Value	HR (95% CI)	*p-*Value
Preoperative PSA	1.02 (1.00–1.04)	**0.047**		
pT	3.88 (2.42–6.22)	**<0.001**	1.80 (1.09–2.96)	**0.022**
RP Gleason score	3.33 (2.48–4.49)	**<0.001**	2.96 (2.16–4.05)	**<0.001**
Margin status	1.00 (0.57–1.74)	0.988		
*Benign adjacent tissue*				
p65 continuous	1.02 (0.94–1.11)	0.630	1.02 (0.96–1.09)	0.500
p65 >3% versus ≤3%	1.13 (0.63–2.01)	0.679	1.25 (0.70–2.22)	0.455
*Tumor tissue*				
p65 continuous	1.04 (1.01–1.06)	**0.003**	1.02 (0.99–1.04)	0.178
p65 >3% versus ≤3%	2.13 (1.23–3.66)	**0.007**	1.82 (1.05–3.16)	**0.033**

Significant *p*-values given in bold.

95% CI, 95% confidence interval; HR, hazard ratio; PSA, prostate-specific antigen; pT, pathological staging of the primary tumor; RP, radical prostatectomy.

### Nuclear expression of p65 is an independent predictor of PC-specific mortality

Overall survival of PC patients was assessed only in the Validation-TMA series since the number of events was too small in the Test-TMA series. The percentage of tumor cells showing nuclear expression of p65 was significantly associated with shorter PC-specific survival (*p* = 0.001) (Fig 2E). Univariate Cox regression analysis showed a significant association of PC-specific mortality with staging (HR 3.26 [95% CI 1.84–5.78], *p* < 0.001) and RP Gleason score (HR 3.56 [95% CI 2.43–5.20], *p* < 0.001) (Table 6). Moreover, dichotomization of nuclear p65 frequency in tumor tissues was also significantly associated with disease-specific mortality (HR 3.12 [95% CI 1.55–6.27], *p* = 0.001), and this association remained independent when combined with clinical parameters (HR 2.63 [95% CI 1.30–5.31], *p* = 0.033). Overall, our results show that p65 nuclear frequency is an independent prognostic marker for PC.

**Table 6:** Univariate and multivariate Cox regression analysis predicting prostate-cancer-specific mortality in the Validation-TMA series.

Parameter	Univariate analysis		Multivariate analysis	
	HR (95% CI)	*p-*Value	HR (95% CI)	*p-*Value
pT	3.26 (1.84–5.78)	**<0.001**	1.46 (0.79–2.68)	0.222
RP Gleason score	3.56 (2.43–5.20)	**<0.001**	3.18 (2.13–4.74)	**<0.001**
*Benign adjacent tissue*				
p65 continuous	1.00 (0.89–1.14)	0.937		
p65 >3% versus ≤3%	1.32 (0.66–2.64)	0.430		
*Tumor tissue*				
p65 continuous	1.02 (0.99–1.06)	0.147		
p65 >3% versus ≤3%	3.12 (1.55–6.27)	**0.001**	2.63 (1.30–5.31)	**0.033**

Significant *p*-values given in bold.

95% CI, 95% confidence interval; HR, hazard ratio; pT, pathological staging of the primary tumor; RP, radical prostatectomy.

## Discussion

Integration of powerful prognostic biomarkers in the pathology workflow would help identify patients with an increased risk of developing an aggressive disease. PSA level, stage, and Gleason score are the main prognostic parameters used to identify low-, intermediate-, and high-risk PC. The accuracy of PC stratification would gain from the addition of new prognostic biomarkers. The use of immunohistochemistry is a particularly suitable approach since immunohistochemistry is already used routinely by genitourinary pathologists. Immunohistochemical assays also allow for evaluation of protein subcellular localization [19]. In particular, the combination of immunohistochemistry and digital pathology could facilitate the standardization of biomarker expression analysis and simplify evaluation [20].

The goal of the CPCBN research program is to identify promising markers for integration into the clinical workflow to improve PC patient management [16]. The pan-Canadian multi-centre cohort is an important addition to PC research to support the validation of potential biomarkers that have been reported from studies using small cohorts from single institutions. Previously, we and others have shown that overall p65 expression, or more specifically its nuclear frequency in PC, is associated with BCR in single institution cohorts [8–14,21–23]. Here, we identified a threshold of p65 nuclear expression in a multi-centre cohort of 250 PC patients and validated this prognostic biomarker in a second independent multi-centre cohort of 1,262 PC patients. This study represents the first report of biomarker evaluation using the Test-TMA and Validation-TMA series of the CPCBN and demonstrates the usefulness of the resource for the biomedical community.

In addition to validating nuclear p65 as a predictor of BCR, we found that this biomarker was also associated with bone metastasis development and PC-specific death. These observations, to our knowledge, have never been reported previously and highlight that such findings require large cohorts with longer follow-up, such as the Validation-TMA series, which includes a median follow-up of 71 months, to achieve statistically significant associations. The present study shows that bone-metastasis-free survival and PC-specific survival are shorter for patients with higher p65 nuclear frequency in cancer cells. This biomarker is an independent predictor of prognosis since it remained significant when analyses were adjusted for pathological staging of the primary tumor and Gleason score at RP. In addition, patients with a high frequency of nuclear p65 expression showed a greater risk for PC-specific death than those with lower expression. Preoperative PSA value as a biomarker failed to reach statistical significance for the prediction of bone metastases and PC mortality. These data suggest that p65 could augment the established clinical prognostic markers used to stratify PC risk and could be a useful parameter in pathological practice.

Our results are compatible with the known activities of the NF-κB pathway. Although activation of NF-κB does not induce the development of PC, its expression is associated with the progression of the disease. The inactivation of IκBα, an inhibitor of p65, in the Hi-Myc mouse PC model increases the aggressiveness of the disease [24]. Moreover, activation of NF-κB pathways in PC cells induces the expression of osteoclastogenic genes such as *receptor activator of NF-κB ligand (RANKL)* and *parathyroid hormone-related protein (PTHrP)* [25]. These 2 proteins are well known for their contribution to the development of bone metastases. Our study shows that nuclear localization of p65 is an independent predictor of bone metastases and disease-specific death. In addition, constitutive activation of p65 has been identified during progression of PC to castration resistance, a stage when patients no longer respond to anti-androgen therapy [26].

We also hypothesize that nuclear p65 could act as a predictive biomarker for specific treatments. Bortezomib is recognized to block NF-κB pathways through the inhibition of the 26S proteasome [27]. This specific mechanism involves inhibition of IκB degradation, confining NF-κB to the cytoplasm. Currently, bortezomib is used to treat cancers such as multiple myeloma and mantle cell lymphoma, in which NF-κB is highly activated. However, the NF-κB pathway blocker is not yet indicated for phase III studies for PC patients due to the inadequate activity-to-toxicity ratio [28–30]. The recruitment of PC patients with a highly activated NF-κB pathway may be necessary to ensure a higher response rate. Future studies should be considered to evaluate the nuclear frequency of p65 by immunohistochemistry on PC tissues from early phase bortezomib trials to evaluate the theragnostic potential for p65 in this treatment regimen.

In conclusion, we demonstrated the prognostic significance of nuclear p65 in 2 independent PC TMA cohorts that included primary tumor tissues of patients from multiple institutions. To our knowledge, our study is the first to highlight the prognostic ability of nuclear p65 to identify patients with an increased risk of developing bone metastases and PC-specific mortality. The implementation of this biomarker in the clinical workflow would allow genitourinary pathologists and clinicians to improve the identification of patients with high-risk PC.

## Supporting information

S1 Table STROBE reporting checklist. (DOCX)

S2 Table Membership of the Canadian Prostate Cancer Biomarker Network. (DOCX)

## Abbreviations

BCR: biochemical recurrence
CPCBN: Canadian Prostate Cancer Biomarker Network
HR: hazard ratio
PC: prostate cancer
PSA: prostate-specific antigen
RP: radical prostatectomy
TMA: tissue microarray
