# Supplementary material for: Validation of the prognostic value of NF-κB p65 in prostate cancer: A retrospective study using a large multi-institutional cohort of the Canadian Prostate Cancer Biomarker Network
Source: PLoS Med. 2019 Jul 2;16(7):e1002847. doi: 10.1371/journal.pmed.1002847 (PMC6605640; doi:10.1371/journal.pmed.1002847)
Supplement: S1 Table — (DOCX) [file pmed.1002847.s001.docx]

**Supplementary Table 1: STROBE reporting checklist** Strobes

|  | **Item No** | **Recommendation** |
| --- | --- | --- |
| **Title and abstract** | 1 | (*a*) Indicate the study’s design with a commonly used term in the title or the abstract |
|  |  | Title and abstract (paragraph of methods and findings) |
|  |  | (*b*) Provide in the abstract an informative and balanced summary of what was done and what was found |
|  |  | Abstract (paragraph of methods and findings) |
| **Introduction** | | |
| Background/rationale | 2 | Explain the scientific background and rationale for the investigation being reported |
|  |  | Introduction (paragraphs 1-4) |
| Objectives | 3 | State specific objectives, including any prespecified hypotheses |
|  |  | Introduction (paragraph 5) |
| **Methods** | | |
| Study design | 4 | Present key elements of study design early in the paper |
|  |  | Methods (Patients and CPCBN TMAs) |
| Setting | 5 | Describe the setting, locations, and relevant dates, including periods of recruitment, exposure, follow-up, and data collection |
|  |  | Methods (Patients and CPCBN TMAs) |
| Participants | 6 | (*a*) Give the eligibility criteria, and the sources and methods of selection of participants. Describe methods of follow-up |
|  |  | Methods (Patients and CPCBN TMAs) |
|  |  | (*b*) For matched studies, give matching criteria and number of exposed and unexposed |
|  |  | N/A |
| Variables | 7 | Clearly define all outcomes, exposures, predictors, potential confounders, and effect modifiers. Give diagnostic criteria, if applicable |
|  |  | Methods (Patients and CPCBN TMAs) |
| Data sources/ measurement | 8* | For each variable of interest, give sources of data and details of methods of assessment (measurement). Describe comparability of assessment methods if there is more than one group |
|  |  | Methods (Patients and CPCBN TMAs) |
| Bias | 9 | Describe any efforts to address potential sources of bias |
|  |  | Methods (Quantification of nuclear p65 expression) |
| Study size | 10 | Explain how the study size was arrived at |
|  |  | Methods (Patients and CPCBN TMAs) |
| Quantitative variables | 11 | Explain how quantitative variables were handled in the analyses. If applicable, describe which groupings were chosen and why |
|  |  | Methods (Quantification of nuclear p65 expression) |
| Statistical methods | 12 | (*a*) Describe all statistical methods, including those used to control for confounding |
|  |  | Methods (Statistics) |
|  |  | (*b*) Describe any methods used to examine subgroups and interactions |
|  |  | Methods (Statistics) |
|  |  | (*c*) Explain how missing data were addressed |
|  |  | Methods (Quantification of nuclear p65 expression, and Statistics) |
|  |  | (*d*) If applicable, explain how loss to follow-up was addressed |
|  |  | Methods (Statistics) |
|  |  | (*e*) Describe any sensitivity analyses |
|  |  | N/A |
| **Results** | | |
| Participants | 13* | (a) Report numbers of individuals at each stage of study—eg numbers potentially eligible, examined for eligibility, confirmed eligible, included in the study, completing follow-up, and analysed |
|  |  | Results (The nuclear localization of p65 in PC and increased risk of BCR, paragraph 1) |
|  |  | (b) Give reasons for non-participation at each stage |
|  |  | N/A |
|  |  | (c) Consider use of a flow diagram |
|  |  | N/A |
| Descriptive data | 14* | (a) Give characteristics of study participants (eg demographic, clinical, social) and information on exposures and potential confounders |
|  |  | Results (Table 1) |
|  |  | (b) Indicate number of participants with missing data for each variable of interest |
|  |  | N/A |
|  |  | (c) Summarise follow-up time (eg, average and total amount) |
|  |  | Results (Table 1) |
| Outcome data | 15* | Report numbers of outcome events or summary measures over time |
|  |  | Results (Table 1) |
| Main results | 16 | (*a*) Give unadjusted estimates and, if applicable, confounder-adjusted estimates and their precision (eg, 95% confidence interval). Make clear which confounders were adjusted for and why they were included |
|  |  | Results (Table 2, 3, 4, 5 and 6) |
|  |  | (*b*) Report category boundaries when continuous variables were categorized |
|  |  | Method (Statistics) and Results (The nuclear localization of p65 in PC and increased risk of BCR, paragraph 2) |
|  |  | (*c*) If relevant, consider translating estimates of relative risk into absolute risk for a meaningful time period |
|  |  | N/A |
| Other analyses | 17 | Report other analyses done—eg analyses of subgroups and interactions, and sensitivity analyses |
|  |  | N/A |
| **Discussion** | | |
| Key results | 18 | Summarise key results with reference to study objectives |
|  |  | Discussion (paragraph 6) |
| Limitations | 19 | Discuss limitations of the study, taking into account sources of potential bias or imprecision. Discuss both direction and magnitude of any potential bias |
|  |  | Abstract (Methods and findings) |
| Interpretation | 20 | Give a cautious overall interpretation of results considering objectives, limitations, multiplicity of analyses, results from similar studies, and other relevant evidence |
|  |  | Discussion (paragraphs 1-5) |
| Generalisability | 21 | Discuss the generalisability (external validity) of the study results |
|  |  | Discussion (paragraph 2) |
| **Other information** | | |
| Funding | 22 | Give the source of funding and the role of the funders for the present study and, if applicable, for the original study on which the present article is based |
|  |  | Acknowledgments |
