# Supplementary material for: Validation of the prognostic value of NF-κB p65 in prostate cancer: A retrospective study using a large multi-institutional cohort of the Canadian Prostate Cancer Biomarker Network
Source: PLoS Med. 2019 Jul 2;16(7):e1002847. doi: 10.1371/journal.pmed.1002847 (PMC6605640; doi:10.1371/journal.pmed.1002847)
Supplement: S2 Table — (DOCX) [file pmed.1002847.s002.docx]

Supplementary Table 2: Membership of The Canadian Prostate Cancer Biomarker Network

| **First Name** | **Last Name** | **Title** | **Email** | **Affiliation** | **Postal Address** | **ORCID** |
| --- | --- | --- | --- | --- | --- | --- |
| Fred | Saad | MD, FRCSC | fredsaad@videotron.ca | Département de Chirurgie, Université de Montréal, Centre de recherche du Centre hospitalier de l'Université de Montréal et Institut du cancer de Montréal | Centre de recherche du CHUM, 900 St-Denis St, Building R, R10.464, Montreal Quebec, H2X0A9 | <https://orcid.org/0000-0003-2986-5617> |
| Anne-Marie | Mes-Masson | PhD, FCAHS, FRSC | anne-marie.mes-masson@umontreal.ca | Département de Médecine, Université de Montréal, Centre de recherche du Centre hospitalier de l'Université de Montréal et Institut du cancer de Montréal | Centre de recherche du CHUM, 900 St-Denis St, Building R, R10.412, Montreal Quebec, H2X0A9 | https://orcid.org/0000-0002-6498-266X |
| Veronique | Ouellet | PhD | veronique.ouellet.chum@ssss.gouv.qc.ca | Centre de recherche du Centre hospitalier de l'Université de Montréal et Institut du cancer de Montréal | Centre de recherche du CHUM, 900 St-Denis St, Building R, R10.416, Montreal Quebec, H2X0A9 | [https://orcid.org/0000-0003-1854-7082](https://orcid.org/0000-0003-1854-7082 ) |
| Dominique | Trudel | MD, FRCSC, PhD | dominique.trudel.chum@ssss.gouv.qc.ca | Département de pathologie et de biologie cellulaire, Université de Montréal, Centre de recherche du Centre hospitalier de l'Université de Montréal et Institut du cancer de Montréal et Institut du cancer de Montréal | Centre de recherche du CHUM, 900 St-Denis St, Building R, R10.440, Montreal Quebec, H2X0A9 | https://orcid.org/0000-0001-9820-1772 |
| Mathieu | Latour | MD, FRCSC | mathieu.latour.chum@ssss.gouv.qc.ca | Département de pathologie et de biologie cellulaire, Université de Montréal et Centre de recherche du Centre hospitalier de l'Université de Montréal | Centre hospitalier de l'Université de Montréal, Department of Pathology, 1051 Sanguinet St, F07.1256, Montreal, Quebec, H2X 3E4 |  |
| Veronique | Barrès | MSC | veronique.barres.chum@ssss.gouv.qc.ca | Centre de recherche du Centre hospitalier de l'Université de Montréal et Institut du cancer de Montréal | Centre de recherche du CHUM, 900 St-Denis St, Building R, R10.214, Montreal Quebec, H2X0A9 |  |
| Nathalie | Delvoye | MSc | nathalie.delvoye.chum@ssss.gouv.qc.ca | Centre de recherche du Centre hospitalier de l'Université de Montréal et Institut du cancer de Montréal | Centre de recherche du CHUM, 900 St-Denis St, Building R, R10.214, Montreal Quebec, H2X0A9 |  |
| Jean-Baptiste | Lattouf | MD, FRCSC | jean-baptiste.lattouf.chum@ssss.gouv.qc.ca | Centre de recherche du Centre hospitalier de l'Université de Montréal et Institut du cancer de Montréal | Centre hospitalier de l'Université de Montréal, Department of Urology 1051 Sanguinet St, Montreal, Quebec, H2X 3E4 | https://orcid.org/0000-0002-8192-0508 |
| Pierre | Karakiewicz | MD, FRCSC | pierre.karakiewicz@umontreal.ca | Cancer Prognostics and Health Outcomes Unit, Centre hospitalier de l’Université de Montréal | Centre hospitalier de l'Université de Montréal, Department of Urology 1051 Sanguinet St, Montreal, Quebec, H2X 3E4 |  |
| Armen | Aprikian | MD, FRCSC | armen.aprikian@muhc.mcgill.ca | Deptartment of Surgery (Urology)  McGill University and Cedars Cancer Centre and McGill University Health Centre | 1001 Boulevard Decarie, D02.8100 Montreal, Quebec, H4A 3J1 |  |
| Fadi | Brimo | MD, FRCSC | fadi.brimo@mcgill.ca | Deptartment of Surgery (Urology)  McGill University and McGill University Health Centre | 1001 Boulevard Decarie, E4. 4188 Montreal, Quebec, H4A 3J1 |  |
| Simone | Chevalier | PhD | simone.chevalier@mcgill.ca | Deptartment of Surgery (Urology)  McGill University and McGill University Health Centre Research Institute | 1001 Boulevard Decarie, E2.2210 Montreal, Quebec, H4A 3J1 | https://orcid.org/0000-0001-7009-0003 |
| Lucie | Hamel | PhD | lucie.hamel@mail.mcgill.ca | McGill University Health Centre Research Institute | 1001 Boulevard Decarie, E2.4380 Montreal, Quebec, H4A 3J1 |  |
| Eleonora | Scarlata | PhD | eleonora.scarlata@mail.mcgill.ca | McGill University Health Centre Research Institute | 1001 Boulevard Decarie, E2.4380 Montreal, Quebec, H4A 3J1 | https://orcid.org/0000-0001-9412-7763 |
| Louis | Lacombe | MD, FRCSC | louis.lacombe@crchudequebec.ulaval.ca | Centre de recherche du Centre hospitalier universitaire de Québec-Université Laval | 10 McMahon, Québec, QC, G1R 3S1 | https://orcid.org/0000-0003-4811-0308 |
| Alain | Bergeron | PhD | alain.bergeron@crchudequebec.ulaval.ca | Centre de recherche du Centre hospitalier universitaire de Québec-Université Laval | 10 McMahon, Québec, QC, G1R 3S1 | https://orcid.org/0000-0002-2875-0773 |
| Hélène | Hovington | BSc | helene.hovington@crchudequebec.ulaval.ca | Centre de recherche du Centre hospitalier universitaire de Québec-Université Laval | 10 McMahon, Québec, QC, G1R 3S1 |  |
| Hervé | Brisson | MSc | hervebrisson1959@gmail.com | Centre de recherche du Centre hospitalier universitaire de Québec-Université Laval | 10 McMahon, Québec, QC, G1R 3S1 |  |
| Céline | Veilleux | Inf. | celine.veilleux@crchudequebec.ulaval.ca | Centre de recherche du Centre hospitalier universitaire de Québec-Université Laval | 10 McMahon, Québec, QC, G1R 3S1 |  |
| Neil E | Fleshner | MD, FRCSC | Neil.Fleshner@uhn.on.ca | Division of Urology, University of Toronto, Princess Margaret Hospital | Princess Margaret Cancer Centre Department of Surgical Oncology 610 University Avenue Toronto, ON M5G 2M9 |  |
| Theodorus | van der Kwast | MD, FRCSC | Theodorus.vanderKwast@uhn.ca | Cancer Clinical Research Unit (CCRU), Princess Margaret Cancer Centre | Department of Pathology Toronto General Hospital 200 Elizabeth St. Rm 11E-220 Toronto Ontario M5G 2C4 | https://orcid.org/0000-0001-8640-5786 |
| Martin | Gleave | MD, FRCSC | m.gleave@ubc.ca | Department of Urologic Sciences,  The Vancouver Prostate Centre and  University of British Columbia | Vancouver Prostate Centre, Jack Bell Research Centre 2660 Oak Street Vancouver, BC V6H 3Z6 | https://orcid.org/0000-0003-4235-0167 |
| Ladan | Fazli | MD, FRCSC | lfazli@prostatecentre.com | The Vancouver Prostate Centre and  University of British Columbia | Vancouver Prostate Centre, Jack Bell Research Centre 2660 Oak Street Vancouver, BC V6H 3Z6 | https://orcid.org/0000-0001-6488-4245 |
